# Moxibustion for Chemotherapy-Induced Nausea and Vomiting: A Systematic Review and Meta-Analysis

**DOI:** 10.1155/2017/9854893

**Published:** 2017-10-12

**Authors:** Ziling Huang, Zongshi Qin, Qin Yao, Yuanxuan Wang, Zhishun Liu

**Affiliations:** ^1^Department of Acupuncture, Guang'anmen Hospital, China Academy of Chinese Medical Sciences, Beijing 100053, China; ^2^Beijing University of Chinese Medicine, Beijing 100029, China

## Abstract

Nausea and vomiting are distressing symptoms for patients receiving chemotherapy. Moxibustion, which involves the use of burning moxa to generate heat and stimulate acupoints, has been reported to potentially ameliorate chemotherapy-induced side effects, particularly nausea and vomiting. This systematic review evaluated current evidence on the effectiveness of moxibustion against chemotherapy-induced nausea and vomiting (CINV). We searched eight online databases and two trial registries for relevant trials. The random-effects model was used to conduct a meta-analysis. Furthermore, the risk ratio (RR) and mean difference (MD) were used to explain dichotomous and continuous outcomes, respectively; the outcomes were within 95% confidence intervals (CIs). The results revealed that moxibustion might more favorably relieve the severity and frequency of CINV, compared with no treatment (RR: 2.04, 95% CI: 1.42–2.93); moxibustion might have stronger effects than antiemetic drugs (RR: 1.87, 95% CI: 1.27–2.76). There is no robust result that moxibustion could enhance the effects of antiemetic drugs administered as a complementary treatment. Actual moxibustion (8.10 ± 10.98) may have more favorable effects than placebo moxibustion (46.67 ± 23.32). However, the evidence obtained is not sufficient because of the lack of strict clinical trials.* Protocol Registration*. This trial is registered with PROSPERO CRD42016030037.

## 1. Introduction

Chemotherapy, a major cancer treatment, is aimed at ameliorating symptoms and prolonging patients' life [1]. However, nausea and vomiting are common side effects of chemotherapy in patients with cancer; these effects are defined as chemotherapy-induced nausea and vomiting (CINV) [2, 3]. Neurotransmitters, such as 5-hydroxytryptamine, substance P, and dopamine, may play an important role in CINV. Treatment- and patient-specific effects are risk factors for CINV [4, 5].

Based on the emetogenic potential of intravenous antineoplastic agents, chemotherapeutic drugs can be classified into four categories: high-emetic-risk (higher than 90%), moderate-emetic-risk (30%–90%), low-emetic-risk (10%–30%), and minimal-emetic-risk (lower than 10%) agents [1, 6]. Considering the duration of nausea and vomiting, CINV can be classified into the following categories: acute CINV, which occurs in a few minutes of chemotherapy and can be relieved in 24 hours; delayed CINV, which occurs after more than 24 hours, typically reaches its peak from 48 to 72 hours, and can last 5 days after chemotherapy; and anticipatory emesis, which may occur when patients see or smell the chemotherapeutic drugs [1, 6]. Many agents are available for preventing CINV, and they include 5-HT3 receptor antagonists, NK1 receptor antagonists, and corticosteroids. Dopamine receptor antagonists, benzodiazepines, olanzapine, and cannabinoids are other alternatives [1, 2, 6–9].

CINV occurs in 70–80% patients treated with chemotherapy [2, 3]. Although antiemetic drugs can ameliorate symptoms to a certain extent, they result in other side effects such as diarrhea, fatigue, headache, and transaminase elevation [6]. However, approximately 30%–60% of patients experience nausea and vomiting despite using antiemetic drugs [2]. CINV can cause anxiety and depression and reduce patients' quality of life. Furthermore, CINV negatively affects 20% patients' will to complete the cancer treatment [2]. Occasionally, patients postpone or refuse to continue chemotherapy because of the mentioned reasons [3, 8, 10]. Therefore, preventing and relieving CINV in patients with cancer have become a major concern in cancer treatment.

Moxibustion, a traditional Chinese medicine treatment, involves the use of burning moxa (dried leaves of an Asian species of mugwort) to generate heat and stimulate acupoints. Direct and indirect moxibustion are the frequently used moxibustion techniques. Direct moxibustion entails igniting a moxa cone or stick directly on acupoints, whereas indirect moxibustion involves using certain types of materials, such as ginger, salt, or herbs, between the ignited moxa cone or stick and acupoints. The treatment is continued until the skin turns red and patients feel warm and comfortable around the acupoints. Moxibustion is a noninvasive, painless, and easily operative treatment with fewer adverse events, and patients can perform it at home. In addition, it is considered a safe and effective complementary treatment [11, 12].

Two systematic reviews, published in Chinese and English languages, have analyzed the effects of moxibustion on chemotherapy-related side effects [12, 13]. These reviews have reported that moxibustion could facilitate alleviating chemotherapy-induced side effects, particularly nausea and vomiting. Nevertheless, drawing an accurate conclusion from these reviews is difficult because of limited evidence. Recent (2005–2017) randomized controlled trials (RCTs) [14–29] have investigated CINV, but without further analysis. Therefore, we conducted this systematic review to determine the effectiveness of moxibustion.

## 2. Methods

### 2.1. Review Question

Can moxibustion relieve the severity and frequency of CINV?

### 2.2. Types of Studies

We included RCTs on the effects of moxibustion on CINV that were published in Chinese and English. Non-RCTs, quasi-randomized trials, reviews, animal trials, or trials without full texts were excluded. Eligible RCTs included those involving randomized control, diagnostic criteria, intervention measures, and statistical methods. The results matched with the criteria used in each RCT.

### 2.3. Types of Patients

Patients diagnosed as having cancer and CINV were included. Differences in age, sex, race, and educational or economic status among patients were disregarded.

Patients with mental disorders, acute infections, and allergy to moxibustion, as well as those with diseases that may cause nausea and vomiting, were excluded.

### 2.4. Types of Interventions

Two types of moxibustion were included: direct and indirect moxibustion. Control interventions included no treatment, antiemetic drugs, and placebo moxibustion.

Comparisons between moxibustion types were excluded. Comparisons of moxibustion with Chinese medicine or acupuncture were also excluded.

### 2.5. Types of Outcome Measures

The primary outcome was the severity and frequency of CINV during chemotherapy. Three criteria were used to evaluate the severity and frequency of nausea and vomiting: the World Health Organization (WHO) criterion for acute and subacute toxicity of anticancer agents, curative effect of Eastern Society for Medical Oncology (ESMO), and score of the European Organization for Research on Treatment of Cancer questionnaire v3.0 (EORTC QLQ-C30 v3.0; the nausea and vomiting domain).

The secondary outcomes were the physical condition and quality of life after chemotherapy. The Karnofsky performance score (KPS) was used to estimate the physical condition, and EORTC QLQ-C30 v3.0 was used to assess quality of life. Adverse events were also evaluated.

### 2.6. Search Methods for Study Identification

We searched the following databases from their inception until February 2017 for RCTs investigating the effects of moxibustion on CINV that have been published in Chinese and English: EMBASE, PubMed, Cochrane Central Register of Controlled Trials, Chinese Biomedical Literature Database, China National Knowledge Infrastructure, Chinese Medical Current Content, Chinese Scientific Journal Database (VIP database), and Wanfang Database. Ongoing and registered trials were searched in two trial registries (https://www.clinicaltrials.gov and http://www.who.int/trialsearch/Default.aspx).

The PubMed database was searched using the search strategy in the Cochrane Handbook for Systematic Reviews of Interventions, Version 5.1.0 [30]. The following keywords were used: (randomized controlled trial OR controlled clinical trial OR randomized OR randomly OR trial OR groups) AND (moxibustion OR moxabustion OR mugwort OR moxa) AND (drug therap^*∗*^ OR chemotherap^*∗*^ OR pharmacotherap^*∗*^) AND (nausea OR vomiting OR emesis). The search strategy for the other online databases was adjusted according to their requirements.

### 2.7. Selection of Studies

The studies selected from the databases were integrated into Endnote X7 (Thomson Reuters, New York, NY, USA). After removing duplicate studies, two authors (Z. H. and Q. Y.) conducted primary screening by reviewing the titles and abstracts of each trial to select eligible trials. They subsequently performed secondary screening by reviewing the full texts of each trial to select eligible trials and further assess them. These steps were separately completed by the two authors. Differences in opinion between the two authors were resolved by a third author (Z. L.).

### 2.8. Extraction and Management of Data

The data from each included trial were extracted and recorded in a data extraction form by two authors (Z. H. and Q. Y.) separately. The following factors were analyzed: general information (publication country, year, language, and author details), participants (baseline characteristics, inclusion criteria, exclusion criteria, and sample size), interventions (type, frequency, and course of moxibustion), comparisons (type, dose, frequency, and course of comparison treatments), outcomes (severity and frequency of nausea and vomiting, score of the included scale and questionnaire), and adverse events. The authors of the studies were contacted, when required. RevMan V.5.3 was used for data analysis.

### 2.9. Assessment of Risk of Bias in Included Studies

The Cochrane Collaboration tool for assessing the risk of bias in randomized trials [31] was used to assess the risk of bias in each included trial. The following evaluations were performed by two authors (Z. Q. and Q. Y.) separately: selection bias (random sequence generation and allocation concealment), performance bias (blinding of the participants and personnel), detection bias (blinding of the outcome assessment), attrition bias (incomplete outcome data), reporting bias, and other sources of bias. We assessed publication bias by using funnel plots; however, this process could not be completed because of the limited number of trials. The third author (Z. L.) made final decisions on this process.

### 2.10. Data Synthesis

We used RevMan V.5.3 for data synthesis. A meta-analysis was performed to analyze the study data, if possible. We used the risk ratio (RR) and mean difference (MD) to explain dichotomous and continuous outcomes, respectively. The outcomes were within 95% confidence intervals (CIs).

Considering the differences in each trial (variations in chemotherapy, moxibustion type, acupoints, and antiemetic drugs), the random-effects model was used to perform the meta-analysis.

The *I*^2^ statistic was used for assessing statistical heterogeneity. We classified the articles with *I*^2^ < 50% as having low heterogeneity, whereas those with *I*^2^ > 75% were classified as having high heterogeneity. We determined the potential factors for heterogeneity by performing a sensitivity analysis. Instead of a meta-analysis, a descriptive analysis was performed when *I*^2^ > 75%.

## 3. Results

### 3.1. Results of the Search

We obtained 272 studies from the online databases; we included 251 studies after removing duplicates. Through primary screening, we ruled out 190 studies by screening their titles and abstract. After reviewing the full texts of 61 studies, we excluded studies because they were not true RCTs (*n* = 4), were duplicates (*n* = 6), lacked adequate data (*n* = 30), conducted comparisons with other traditional Chinese medicine treatments (*n* = 2), were case reports (*n* = 1), and had no full text (*n* = 2). Finally, 16 trials matched our study criteria and were included in this systematic review and meta-analysis [14–29]. These trials were published in Chinese during September 2005 and February 2017. One trial [28] was conducted in Mongolia, whereas others have been conducted in China. Two trials [18, 26] were determined to be multicenter trials, whereas the rest were determined to be single-center trials.

**Figure 1:**
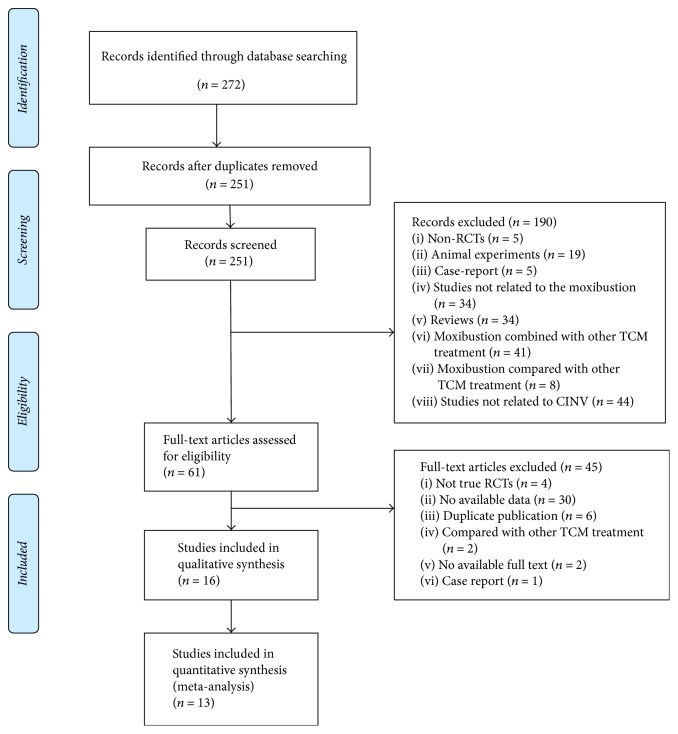
Flowchart of study selection.

Figure 1 summarizes the progress of study selection.

### 3.2. Patients

We included a small sample of 1123 patients (age: 20–80 years) from the 16 trials in our systematic review. Patients in all trials were diagnosed as having cancer and received chemotherapy. Patients were diagnosed as having lung cancer in three trials (*n* = 170) [19, 24, 29], breast cancer in one trial (*n* = 58) [23], colon cancer in one trial (*n* = 60) [21], and gastric cancer in one trial (*n* = 91) [18]. The remaining patients (*n* = 744) were diagnosed as having various other cancers (esophageal cancer, ovarian cancer, liver cancer, and nasopharyngeal cancer).

### 3.3. Interventions of Moxibustion

Practitioners typically select acupoints for moxibustion based on traditional Chinese medicine theories and clinical experience. Ten trials used direct moxibustion: wheat moxibustion (*n* = 1) [14], moxa box moxibustion (*n* = 1) [27], cone moxibustion (*n* = 1) [17, 19, 24, 28, 32], and stick moxibustion (*n* = 3) [15, 20, 21]. The remaining six trials used indirect moxibustion: ginger-partitioned moxibustion (*n* = 5) [16, 22, 23, 25, 26] and herb-partitioned moxibustion (*n* = 1) [18]. Zusanli (ST36; 10/16, 62.50%), Zhongwan (RN12; 6/16, 37.50%), Geshu (BL17; 5/16, 31.25%), and Danshu (BL19; 4/16, 25.00%) were the frequently used acupoints. Moxibustion was performed before the chemotherapy course in three trials [21, 22, 26], whereas in the remaining trials, it was conducted during the chemotherapy course. Moxibustion was typically operated until the patients' local skin became warm and turned red. Moxibustion was conducted once daily (*n* = 12), twice daily (*n* = 2), three times weekly (*n* = 1), and six times weekly (*n* = 1).

### 3.4. Control Interventions

Five trials compared moxibustion with antiemetic drugs [14, 17, 18, 21, 23], and seven compared moxibustion plus antiemetic drugs with the same antiemetic drugs [15, 16, 20, 24, 27–29]. The antiemetic drugs used in these 12 trials belong to the 5-HT3 receptor antagonist family. Three trials compared moxibustion with no treatment [19, 25, 26]. One trial compared actual moxibustion with placebo moxibustion (a thick paper between the moxa and acupoints) on the same acupoints [22]. One trial [18] did not mention the control intervention in the English abstract, the details of control group can be found in the full text. Three trials [21, 23, 24] did not have English abstract, the details of intervention and control intervention can be found in the full text.

### 3.5. Outcome Measures

The primary outcome was the severity and frequency of CINV in complete response during the chemotherapy course. Based on the American Society of Clinical Oncology (ASCO) guidelines and relevant studies [1, 32, 33], complete response was described as no nausea or vomiting and no use of emergency antiemetic drugs after chemotherapy; complete response matched grade 0 of the WHO and ESMO criteria. Three trials [23, 25, 28] reported the scores in the nausea and vomiting domain of EORTC QLQ-C30 v3.0, whereas the remaining studies reported the grade of nausea and vomiting according to the WHO or ESMO criterion. All outcomes of the included trials were assessed during the chemotherapy course.

Table 1 summarizes the characteristics of each trial.

### 3.6. Risk of Bias

Randomization was conducted in all 16 RCTs. Nine trials used a table of random numbers [14–16, 18, 22, 23, 25, 26, 29], and one used JMTJFX software to generate random numbers [19]. Details on the randomization methods of six trials could not be obtained despite contacting the authors. Only three trials reported allocation concealment [22, 23, 25]. However, these three trials were considered as having an unclear risk of selection bias because of the lack of an indication of whether the envelope was sealed and opaque. Two trials were considered as having a high risk of reporting bias; one study [23] lacked final KPSs after chemotherapy and the other [26] lacked data on supportive treatment and emergency antiemetic drug use. Only one trial [22] used a patient-blinded method, whereas others did not, because of the characteristics of moxibustion. Three trials [14, 15, 23] reported 11 dropouts, and only one of them [15] provided details on their dropouts. Three trials [20, 22, 27] were considered as having an unclear risk of other sources of bias because of the lack of data on consistent baseline characteristics.

Table 2 summarizes the risk of bias in the included studies.

### 3.7. Effects of Moxibustion versus No Treatment

#### 3.7.1. Primary Outcomes

**Figure 2:**
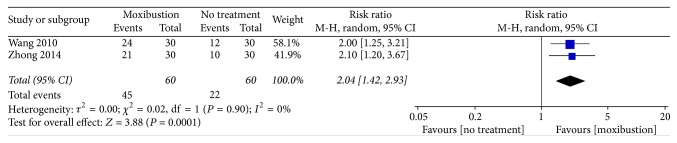
Forest plot of the effects of moxibustion on relieving the severity of CINV (measured using the WHO criterion) compared with the effects of no treatment.

Nausea and vomiting were assessed using WHO criterion. Two trials (*n* = 120) [19, 26] reported that moxibustion might more effectively relieve the severity of CINV, compared with no treatment (RR: 2.04, 95% CI: 1.42–2.93, *n* = 120, and *I*^2^ = 0%, low-quality evidence; Figure 2).

*Nausea and Vomiting Assessed Using EORTC QLQ-C30 v3.0.* One trial [25] revealed that moxibustion might effectively relieve the severity of CINV, compared with no treatment (23.73 ± 19.93 versus 37.73 ± 25.19).

#### 3.7.2. Secondary Outcomes

**Figure 3:**
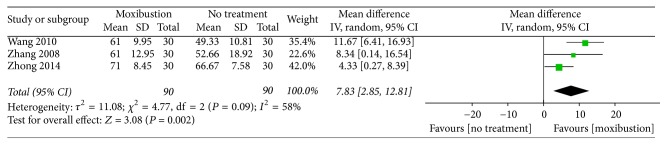
Forest plot of the effects of moxibustion on improving the physical condition (measured using the KPS) compared with the effects of no treatment.

**Figure 4:**
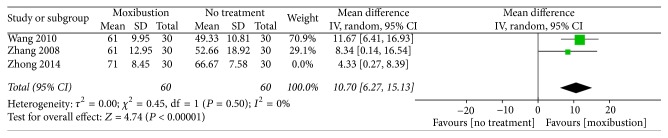
Forest plot of the effects of moxibustion on improving physical condition (measured using the KPS) compared with the effects of no treatment, after the elimination of the trial by Zhong [26].

Physical condition assessed using the KPS. A combination of three trials [19, 25, 26] revealed statistical heterogeneity (MD: 7.83, 95% CI: 2.85–12.81, *n* = 180, and *I*^2^ = 58%, low-quality evidence; Figure 3). After the trial by Zhong [26] was removed, the heterogeneity decreased to 0% (Figure 4). The three trials revealed that, compared with no treatment, moxibustion may improve the physical condition of patients.

#### 3.7.3. Adverse Events

Two trials [19, 25] reported no adverse events in the intervention or control group.

### 3.8. Effects of Moxibustion versus Antiemetic Drugs

#### 3.8.1. Primary Outcomes

**Figure 5:**
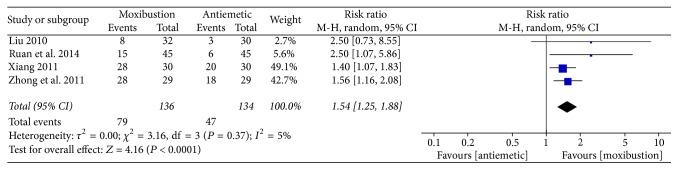
Forest plot of the effects of moxibustion on relieving the severity of CINV (measured using the WHO criterion) compared with antiemetic drugs.

Nausea and vomiting were assessed using the WHO criterion. Four trials [17, 18, 21, 23] revealed that moxibustion may more favorably relieve the severity of CINV, compared with antiemetic drugs (RR: 1.54, 95% CI: 1.25–1.88, *n* = 270, and *I*^2^ = 5%, low-quality evidence; Figure 5).

*Nausea and Vomiting Assessed Using the ESMO Criterion.* One trial [14] that measured outcomes using the ESMO criterion 1996 reported that moxibustion may have more favorable effects on relieve the severity and frequency of CINV than antiemetic drugs (19/27 versus 9/29).

#### 3.8.2. Secondary Outcomes

**Figure 6:**
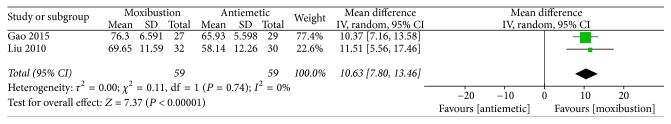
Forest plot of the effects of moxibustion on improving physical condition (measured using the KPS) compared with the effects of antiemetic drugs.

Physical condition assessed using the KPS. Two trials [14, 17] revealed that moxibustion may more effectively improve the physical condition of patients than antiemetic drugs (MD: 10.63, 95% CI: 7.80–13.46, *n* = 118, and *I*^2^ = 0%, low-quality evidence; Figure 6).

#### 3.8.3. Adverse Events

Trials in this group did not report any adverse events in the intervention or control group.

### 3.9. Effects of Moxibustion Plus Antiemetic Drugs versus Antiemetic Drugs Alone

#### 3.9.1. Primary Outcomes

**Figure 7:**
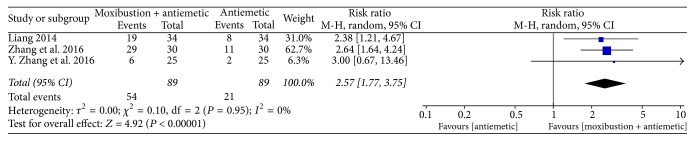
Forest plot of the effects of moxibustion plus antiemetic drugs on relieving the severity of CINV (measured using the WHO criterion) compared with the effects of antiemetic drugs alone.

Nausea and vomiting were assessed using the WHO criterion. Three trials [16, 24, 29] revealed that moxibustion might enhance the effects of antiemetic drugs as a complementary treatment (RR: 2.57, 95% CI: 1.77–3.75, *n* = 178, and *I*^2^ = 0%, low-quality evidence; Figure 7).

**Figure 8:**
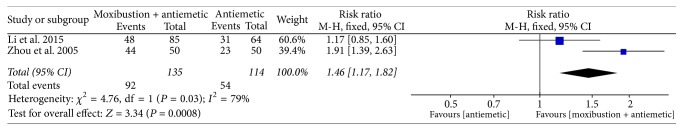
Forest plot of the effects of moxibustion plus antiemetic drugs on relieving the severity and frequency of CINV (measured using the EMSO criterion 1990) compared with the effects of antiemetic drugs alone.

*Nausea and Vomiting Assessed Using the ESMO Criterion.* Two trials measured using the EMSO criterion 1990 [20, 27] with statistical heterogeneity revealed that moxibustion could not enhance the effects of antiemetic drugs as a complementary treatment (MD: 1.49, 95% CI: 0.92–2.43, *n* = 249, and *I*^2^ = 79%, low-quality evidence; Figure 8). One trial measured using the ESMO criterion 1996 [15] reported that moxibustion may enhance the effects of antiemetic drugs as a complementary treatment (complete response; 5/31 versus 2/30).

*Nausea and Vomiting Assessed Using EORTC QLQ-C30 v3.0.* One trial [28] reported that moxibustion may enhance the effects of antiemetic drugs as a complementary treatment and yield an increased score in the nausea and vomiting domain (complete response; 24.5 ± 30.0 versus 36.3 ± 26.7).

#### 3.9.2. Secondary Outcomes

**Figure 9:**
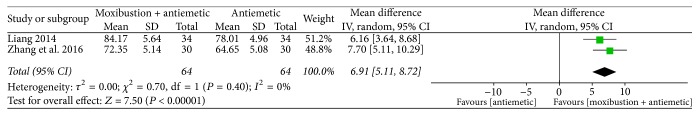
Forest plot of the effects of moxibustion plus antiemetic drugs on improving physical condition (measured using the KPS) compared with the effects of antiemetic drugs alone.

*Physical Condition Assessed Using the KPS*. Two trials [16, 24] reported that moxibustion as a complementary treatment may facilitate improving the physical condition (MD: 6.91, 95% CI: 5.11–8.72, *n* = 128, and *I*^2^ = 0%, low-quality evidence; Figure 9).

*Quality of Life Assessed Using EORTC QLQ-C30 v3.0*. One trial [15] reported that moxibustion as a complementary treatment may facilitate improving the functional score of patients (32.20 ± 7.74 versus 38.39 ± 7.05). One trial [28] reported that moxibustion may increase the quality of life score (56.93 ± 18.6 versus 63.3 ± 22.1).

#### 3.9.3. Adverse Events

Trials in this group did not report any adverse events in the intervention or control group.

### 3.10. Effects of Actual Moxibustion versus Placebo Moxibustion

One trial [22] reported that actual moxibustion might more effectively reduce the score in the nausea and vomiting domain of EORTC QLQ-C30 v3.0 than placebo moxibustion (8.10 ± 10.98 versus 46.67 ± 23.32). Moxibustion could also improve the quality of life score in EORTC QLQ-C30 v3.0 (77.38 ± 10.62 versus 62.50 ± 23.34) and the KPS (80.86 ± 8.87 versus 70.00 ± 11.70). Moreover, this trial did not report any adverse events in the intervention or control group.

## 4. Discussion

This systematic review evaluated the effectiveness of moxibustion against CINV. Among trials comparing moxibustion with no treatment, two [19, 26] reported that moxibustion might more favorably relieve the severity of CINV. These trials had the following similarities: first, patients were diagnosed as having stage III or IV lung cancer; second, moxibustion was performed at the same acupoints (BL17 and BL19); third, cis-platinum was used as the chemotherapeutic drug. The results suggest that moxibustion at the BL17 and BL19 acupoints can alleviate CINV in patients with lung cancer. Zhong's trial [26] was considered as having high reporting bias; this is because although he reported that some patients may have used supportive treatment and ondansetron for severe vomiting, he did not specify who these patients are. A trial [25] that conducted assessments using EORTC QLQ-C30 v3.0 reported that moxibustion might effectively relieve the severity of CINV, but a meta-analysis could not be conducted on a single trial. Moreover, the sample size of this trial was too small to provide strong evidence.

Among the trials comparing actual moxibustion with placebo moxibustion, one [22] showed that actual moxibustion might be more effective than placebo moxibustion in relieving the severity of CINV. Despite no meta-analysis of this trial, the result may still suggest that actual moxibustion has specific effects on CINV. This trial has two sources of bias. First, because patient blinding was not assessed in this trial, it could not estimate whether insulating the heat by using a thick paper successfully blinded the patients. Second, the sample size of this trial was too small to confirm the effectiveness of actual moxibustion. Therefore, the aforementioned suggestion is unreliable.

In conclusion, expectation effects were not determined in trials comparing moxibustion with no treatment and placebo moxibustion. The positive response might be because of the expectation or placebo effects of moxibustion. Thus, because of the low quality of the included trials, we could not obtain robust results on the effectiveness of moxibustion in relieving the severity and frequency of CINV.

Serotonin (5-HT3) receptor antagonists were recommended according to the National Comprehensive Cancer Network guidelines in Oncology-Antiemesis Version 2.2014 [6] and ASCO guideline for antiemetic in oncology update 2006 [1]. According to the ASCO guideline, five 5-HT3 receptor antagonists (dolasetron, granisetron, ondansetron, palonosetron, and tropisetron) have equivalent efficacy at the same doses. One meta-analysis [34] revealed no difference among ondansetron, granisetron, and dolasetron mesylate. Among the trials comparing moxibustion with antiemetic drugs, four trials [17, 18, 21, 23] measured the outcomes by using the WHO criterion. The results with acceptable heterogeneity level showed that moxibustion may more favorably relieve the severity of CINV. One trial [14], measuring the outcomes by using the ESMO criterion 1996, reported that moxibustion may be more effective than antiemetic drugs in relieving CINV. In these five RCTs, 5-HT antagonists were used as the antiemetic drugs (ondansetron in three trials [17, 18, 21], granisetron in one trial [23], and tropisetron in one trial [14]); therefore, the results suggested more favorable effects of moxibustion than of 5-HT receptor antagonists. However, the results are not adequately reliable because of the low quality of the included trials.

Among the studies comparing moxibustion plus antiemetic drugs with antiemetic drugs alone, three RCTs [16, 24, 29] showed that moxibustion might enhance the effects of antiemetic drugs. Two trials [20, 27] measured the outcomes by using the EMSO criterion 1990 with high heterogeneity level revealing that moxibustion could not enhance the effects of antiemetic drugs. The heterogeneity of the two trials may be related to the various types of cancer. The remaining two trials measured outcomes by using EMSO criterion 1996 [15] and EORTC QLQ-C30 v3.0 [28]. These two trials also reported that moxibustion might enhance the effects of antiemetic drugs, but a meta-analysis could not be conducted because these trials had different criteria. The results of these six trials were not consistent; thus we could not obtain robust results on the effectiveness of moxibustion in enhancing the effects of antiemetic drugs as a complementary treatment. Additional trials on CINV are warranted to confirm the results.

For secondary outcomes, among the trials comparing moxibustion with no treatment, three [19, 25, 26] reported that moxibustion might improve the physical condition of patients, as measured using the KPS. We considered that the heterogeneity was engendered by Zhong's trial, because of the supportive treatment or ondansetron. Among the trials comparing actual moxibustion with placebo moxibustion, one [22] reported that moxibustion might be more effective than placebo moxibustion in improving the physical condition and quality of life of patients. However, because of the heterogeneity of these trials, the aforementioned result is not adequately reliable.

In the comparison of moxibustion with antiemetic drugs, two trials [14, 17] showed that moxibustion could more effectively improve the physical condition of patients than antiemetic drugs. More relevant trials are warranted to confirm the results because of the low quality and small sample size of the above trials.

Regarding the comparison of moxibustion plus antiemetic drugs with antiemetic drugs alone, two trials revealed that moxibustion, as a complementary treatment, may facilitate improving the physical condition of patients, as measured using the KPS [16, 24]. The remaining two trials, measured using EORTC QLQ-C30 v3.0 [15, 28], showed that moxibustion, as a complementary treatment, may facilitate improving the physical condition and quality of life of patients. A meta-analysis could not be conducted because these two trials did not apply the same domain of EORTC QLQ-C30 v3.0 criterion.

In conclusion, although the results of the included trials were consistent, the quality of the trials was low. Therefore, the results are not adequately reliable.

Moxibustion may induce few adverse events. Two trials [19, 25] reported no adverse events in the intervention or control group. Moreover, other trials did not report any adverse events. Therefore, based on current evidence, moxibustion is a safe treatment.

All the included trials had two sources of bias. First, the included RCTs used a poor methodology and were not registered in the clinical trial registry. Most trials did not mention the procedures of randomization, allocation concealment, and blinding of statisticians. No trial reported the method of sample size calculation. Therefore, the evidence level of this systematic review is low. Second, all trials were published in Chinese, and most trials were conducted in China. Therefore, definite conclusions for other countries and races could not be derived.

This review has some limitations. First, funnel plots could not detect reporting bias because of the limited number of trials. Second, patient blinding method was not rigorous in the included trial. Suitable sham control device is warranted to further trials. A new moxa device for sham treatment developed by Zhao [35] might be a choice. Furthermore, we could not determine expectation effects because no trials assessed the expectation effects of moxibustion. Third, several reasons affected the results, including differences in the type, frequency, acupoints, and course of moxibustion and course of chemotherapy in each trial. Finally, we included RCTs published only in Chinese and English because of language barriers; thus, we may have missed some high-quality trials published in other languages.

## 5. Conclusion

Moxibustion might effectively relieve the severity and frequency of CINV; however, the expectation effects and the effects of placebo moxibustion cannot be excluded. Moxibustion might be more effective than antiemetic drugs. There is no robust result that moxibustion could enhance the effects of antiemetic drugs as a complementary treatment because of inconsistent results. It might also improve the physical condition and quality of life of patients with cancer. In addition, current evidence reveals that moxibustion is a safe treatment with few adverse events. However, the conclusion of this review is limited because of the lack of high-quality RCTs; further evidence is warranted to update this systematic review.

## Figures and Tables

**Table 1:** Characteristics of each trial.

Study	Design	Sample size	Intervention type	Control group	Moxibustion regimen	Outcome measure	Adverse events
Zhong 2014 [26]	Parallel, 2 arms	60 (30/30)	Ginger-partitioned moxibustion	Supportive treatment; ondansetron only in serious vomiting occurs	From 3 days before to 14 days after chemotherapy, qd	WHO; KPS	Not reported
Zhang 2008 [25]	Parallel, 3 arms	90 (30/30/30)	Ginger-partitioned moxibustion	No treatment	During the chemotherapy, qd	EORTC QLQ-C30 v3.0; KPS	No adverse events in both groups
Zhang 2016 [24]	Parallel, 2 arms	60 (30/30)	Direct moxibustion	Conventional dosage of ondansetron	Once a day for 7 days	WHO; KPS	Not reported
Ruan 2014 [18]	Parallel, 2 arms	91 (45/46)	Herb-partitioned moxibustion	Ondansetron 8 mg, dexamethasone 5 mg, day 1 to day 3, qd omeprazole 40 mg day 4 to day 7, qd metoclopramide 10 mg, tid	Once a day for 14 days	WHO	Not reported
Zhong 2011 [23]	Parallel, 2 arms	58 (29/29)	Ginger-partitioned moxibustion	Granisetron hydrochloride 3 mg, before and after chemotherapy	Once a day for 3 days	WHO	Not reported
Xiang 2011 [21]	Parallel, 2 arms	60 (30/30)	Direct moxibustion	Ondansetron hydrochloride 8 mg, before chemotherapy	1 day before chemotherapy to 3 days after chemotherapy	WHO	Not reported
Wang 2010 [19]	Parallel, 2 arms	60 (30/30)	Direct moxibustion	No treatment	During the chemotherapy, qd	WHO; KPS	No adverse events in both groups
Zhou 2005 [20]	Parallel, 2 arms	100 (50/50)	Direct moxibustion	Granisetron hydrochloride 30 mg, before chemotherapy	Twice a day for 7 days	ESMO	Not reported
Xu 2014 [22]	Parallel, 2 arms	50 (25/25)	Ginger-partitioned moxibustion	Placebo moxibustion	From 5 days before to 60 days after chemotherapy, 3 times a week	EORTC QLQ-C30 v3.0; KPS	Not reported
Liu 2010 [17]	Parallel, 2 arms	62 (32/30)	Direct moxibustion	Conventional dosage of dexamethasone and ondansetron	Once a day for 7 days	WHO; KPS	Not reported
Liang 2014 [16]	Parallel, 2 arms	68 (34/34)	Ginger-partitioned moxibustion	Ranitidine 150 mg, granisetron 3 mg, and nutritional support	Once a day for 63 days	WHO; KPS	Not reported
Hao 2014 [15]	Parallel, 2 arms	61 (31/30)	Direct moxibustion	Conventional dosage of dexamethasone, tropisetron, promethazine, and cimetidine	During the chemotherapy, qd	ESMO; EORTC QLQ-C30 v3.0	Not reported
Gao 2015 [14]	Parallel, 2 arms	60 (30/30)	Direct moxibustion	Conventional dosage of dexamethasone, tropisetron, and lansoprazole	During the chemotherapy, 6 times a week	ESMO; KPS	Not reported
Li 2015 [27]	Parallel, 2 arms	169 (85/84)	Moxa box moxibustion	Tropisetron hydrochloride 5 mg	Twice a day for 8 days	ESMO	Not reported
Ya 2010 [28]	Parallel, 2 arms	24 (12/12)	Direct moxibustion	Conventional dosage of dexamethasone and ondansetron	Once a day for 5 days	EORTC QLQ-C30 v3.0	Not reported
Zhang 2016 [29]	Parallel, 2 arms	50 (25/25)	Direct moxibustion	Tropisetron 5 mg before chemotherapy	Once a day for 6 days	WHO	Not reported

*Notes*. WHO: WHO criterion for the acute and subacute toxicity of anticancer agents, KPS: Karnofsky performance score, EORTC QLQ-C30 v3.0: the European Organization for Research on Treatment of Cancer's questionnaire to assess the quality of life v3.0, and ESMO: the Eastern Society for Medical Oncology criterion.

**Table 2:** Risk of bias for included trials.

Study	Sequence generation	Allocation concealment	Blinding	Incomplete outcome data	Selective outcome reporting	Other sources of bias
Zhong 2014 [26]	(−)	0	0	(−)	(+)	(−)
Zhang 2008 [25]	(−)	0	0	(−)	(−)	(−)
Zhang 2016 [24]	0	0	0	(−)	(−)	(−)
Ruan 2014 [18]	(−)	0	0	(−)	(−)	(−)
Zhong 2011 [23]	(−)	0	0	(−)	(+)	(−)
Xiang 2011 [21]	0	0	0	(−)	(−)	(−)
Wang 2010 [19]	(−)	0	0	(−)	(−)	(−)
Zhou 2005 [20]	0	0	0	(−)	(−)	0
Xu 2014 [22]	(−)	0	0	(−)	(−)	0
Liu 2010 [17]	0	0	0	(−)	(−)	(−)
Liang 2014 [16]	(−)	0	0	(−)	(−)	(−)
Hao 2014 [15]	(−)	0	0	(−)	(−)	(−)
Gao 2015 [14]	(−)	0	0	(−)	(−)	(−)
Li 2015 [27]	0	0	0	(−)	(−)	0
Ya 2010 [28]	0	0	0	(−)	(−)	(−)
Zhang 2016 [29]	(−)	0	0	(−)	(−)	(−)

*Note*. Low risk of bias: (−); unclear: 0; high risk of bias: (+).

## Abbreviations

CINV: Chemotherapy-induced nausea and vomiting
RR: Risk ratio
MD: Mean difference
CIs: Confidence intervals
RCTs: Randomized controlled trials
WHO: World Health Organization
ESMO: Eastern Society for Medical Oncology
EORTC QLQ-C30 v3.0: European Organization for Research on Treatment of Cancer questionnaire v3.0
KPS: Karnofsky performance score
ASCO: American Society of Clinical Oncology.

## References

[1] Oncology ASCO (2006). American Society of Clinical Oncology guideline for antiemetic in oncology: update 2006. *Journal of Clinical Oncology*.

[2] Jordan K., Gralla R., Jahn F., Molassiotis A. (2014). International antiemetic guidelines on chemotherapy induced nausea and vomiting (CINV): content and implementation in daily routine practice. *European Journal of Pharmacology*.

[3] Tricco A. C., Blondal E., Veroniki A. A. (2016). Comparative safety and effectiveness of serotonin receptor antagonists in patients undergoing chemotherapy: A systematic review and network meta-analysis. *BMC Medicine*.

[4] Moradian S., Howell D. (2015). Prevention and management of chemotherapy-induced nausea and vomiting. *International Journal of Palliative Nursing*.

[5] Tageja N., Groninger H. (2014). Chemotherapy-induced nausea and vomiting #285. *Journal of Palliative Medicine*.

[6] National Comprehensive Cancer Network NCCN clinical practice guidelines in Oncology. Antiemesis. https://www.nccn.org/professionals/physician_gls/PDF/antiemesis.pdf.

[7] Gralla R. J., Osoba D., Kris M. G. (1999). Recommendations for the use of antiemetics: evidence-based, clinical practice guidelines. *Journal of Clinical Oncology*.

[8] Janelsins M. C., Tejani M. A., Kamen C., Peoples A. R., Mustian K. M., Morrow G. R. (2013). Current pharmacotherapy for chemotherapy-induced nausea and vomiting in cancer patients. *Expert Opinion on Pharmacotherapy*.

[9] Herrstedt J. (2007). Chemotherapy-induced nausea and vomiting: ESMO Clinical Recommendations for prophylaxis. *Annals of Oncology*.

[10] Rao K. V., Faso A. (2012). Chemotherapy-induced nausea and vomiting: Optimizing prevention and management. *American Health and Drug Benefits*.

[11] Xu J., Deng H., Shen X. (2014). Safety of moxibustion: a systematic review of case reports. *Evidence-Based Complementary and Alternative Medicine*.

[12] Lee M. S., Choi T.-Y., Park J.-E., Lee S.-S., Ernst E. (2010). Moxibustion for cancer care: A systematic review and meta-analysis. *BMC Cancer*.

[13] Yuyu H., Zhai X. Moxibustion for cancer-related symptoms: a systematic review and meta-analysis.

[14] Gao S. (2015). *The clinical research on myelosuppression after chemotherapy treats by wheat moxibustion*.

[15] Hao Z. (2014). *Clinical observation on immune function in patients with mild moxibustion cancer chemotherapy*.

[16] Liang J. (2014). Efficacy of moxibustion with ginger in alleviating side effects of lung chemotherapy. *Guangming Journal of Chinese Medicine*.

[17] Liu S. (2010). *Clinical study on the efficiency of direct contact moxibustion on toxicity and side effects resulted from chemotherapy*.

[18] Ruan Y., Zhang W. X., Xa W., Gu Q., Jiao J., Liu L. (2014). Effect of herb-partitioned moxibustion on chemotherapy-induced nausea and vomiting of gastric cancer. *Chinese Archives of Traditional Chinese Medicine*.

[19] Wang T. (2010). *The impact on the toxic reaction of the chemotherapeutics by direct moxibustion on four flowers acupoint*.

[20] Zhou W., Kao J., Yu C., Ma X. (2005). Clinical observation on moxibusion at zusanli point to treat delayed vomiting caused by Cisplatin. *Journal of Clinical Acupuncture and Moxibustion*.

[21] Xiang P. (2011). The clinical study of moxibustion alleviating nausea and vomiting by colon chemoradiotherapy. *Journal of Emergency in Traditional Chinese Medicine*.

[22] Xu S. A., Yang J., Zhao B. (2014). Possible Therapeutic effect of ginger-partitioned moxibustion on chemotherapy induced toxic side effects and quality of life scale: a clinical observation. *Global Traditional Chinese Medicine*.

[23] Zhong S., Xu S., Xu B., Sun Y., Tian H. (2011). Clinical Study on Moxibustion Combined with Granisetron Hydrochloride Injection in the Treatment of Nausea and Vomiting Caused by Chemotherapy of Breast Cancer. *Journal of Chinese Medicinal Materials*.

[24] Zhang Q., Li L., Fan D. (2016). Effect of Direct Moxibustion and Four - flower Point on Quality of Life of Chemotherapy Patients with Lung Cancer. *Journal of Practical Traditional Chinese Medicine*.

[25] Zhang X. (2008). *Efficacy of moxibustion with ginger in preventing toxicity of chemotherapy and effect on quality of life on cancer*.

[26] Zhong Z. (2014). *Clinical study of treating side effects of chemotherapy of lung cancer by ginger moxibustion on Sihua acupoint*.

[27] Li Q., Zhong M., Ye X., Tang R., Tan X., Li R. (2015). Investigation of vomiting-stopping efficacy of moxa box moxibustion in chemotherapy of platinum-based drugs. *Clinical Journal of Chinese Medicine*.

[28] Ya E. H. T. (2010). *Clinical and experimental studies of moxa at guanyuan on effects of the chemotherapy patients vital signs*.

[29] Zhang Y., Kong T., Zhou H. (2016). Moxibustion in treatment of gastrointestinal adverse effects of patients with non-small cell lung cancer in chemotherapy. *Journal of Chinese Medicine*.

[30] Higgins J. P., Green S. (2011). *Cochrane Handbook for Systematic Reviews of Interventions Version 5.1.0*.

[31] Higgins J. P. T., Altman D. G., Gøtzsche P. C. (2011). The Cochrane Collaboration's tool for assessing risk of bias in randomised trials. *The British Medical Journal*.

[32] Schwartzberg L. S., Modiano M. R., Rapoport B. L. (2015). Safety and efficacy of rolapitant for prevention of chemotherapy-induced nausea and vomiting after administration of moderately emetogenic chemotherapy or anthracycline and cyclophosphamide regimens in patients with cancer: A randomised, active-controlled, double-blind, phase 3 trial. *The Lancet Oncology*.

[33] Kim J. E., Hong Y. S., Lee J.-L. (2015). A randomized study of the efficacy and safety of transdermal granisetron in the control of nausea and vomiting induced by moderately emetogenic chemotherapy in Korean patients. *Supportive Care in Cancer*.

[34] Barton D. L., Thanarajasingam G., Sloan J. A. (2014). Phase III double-blind, placebo-controlled study of gabapentin for the prevention of delayed chemotherapy-induced nausea and vomiting in patients receiving highly emetogenic chemotherapy, NCCTG N08C3 (Alliance). *Cancer*.

[35] Zhao B.-X., Chen H.-Y., Shen X.-Y., Lao L. (2014). Can moxibustion, an ancient treatment modality, be evaluated in a double-blind randomized controlled trial? - A narrative review. *Journal of Chinese Integrative Medicine*.

